# Exosomes in Traumatic Brain Injury: From Secondary Injury Mechanisms to Cell‐Free Neurorepair

**DOI:** 10.1002/brb3.71897

**Published:** 2026-11-03

**Authors:** Guiling Gu, Hailiang Tang, Haixia Fan

**Affiliations:** ^1^ Department of Rehabilitation The First People's Hospital of Chenzhou Chenzhou Hunan Province China; ^2^ Department of Neurology The First Affiliated Hospital of Jinan University Guangdong China; ^3^ Department of Neurology First Hospital of Shanxi Medical University Taiyuan Shanxi Province China

**Keywords:** bibliometric analysis, cell‐free therapy, exosomes, neuroinflammation, secondary injury, traumatic brain injury

## Abstract

**Background:**

Traumatic brain injury (TBI) is a leading cause of death and long‐term neurological disability worldwide. Exosomes (EXOs) have attracted increasing attention because they mediate intercellular communication and show promise as diagnostic biomarkers and cell‐free therapeutics. However, the overall research landscape and evolving priorities of exosomes studies in TBI remain insufficiently characterized.

**Methods:**

We performed a bibliometric analysis of publications indexed in the Web of Science Core Collection (WoSCC) and Scopus (search date: October 24, 2025) using equivalent search strategies combining traumatic brain injury and exosome/extracellular vesicle terms. English‐language original research and review articles published between January 1, 2012 and October 24, 2025 were eligible; editorials, letters, conference abstracts, and non‐English publications were excluded. After deduplication and screening, 609 publications were retained. A multi‐tool analytical framework was employed: CiteSpace (v6.4.R1) for citation‐burst detection, co‐occurrence clustering, and timeline visualization; VOSviewer (v1.6.18) for collaboration network mapping and keyword co‐occurrence analysis; and the Bibliometrix R package (v4.4.3) for descriptive statistics, productivity indicators (h‐index, g‐index, m‐index), and performance metrics.

**Results:**

From 2012 to 2025, research on exosomes in TBI expanded steadily, with accelerated growth after 2017. Keyword co‐occurrence, citation‐burst detection, and reference co‐citation analyses showed a clear thematic evolution. Early studies and highly co‐cited references primarily addressed secondary‐injury mechanisms, particularly neuroinflammation, microglial activation, glial–neuronal interactions, and blood–brain barrier dysfunction. Influential work by frequently co‐cited authors (e.g., Chopp M and Zhang Y) helped establish exosomes‐mediated neuroprotection and neurovascular remodeling as central themes. More recently, burst keywords and emerging co‐citation clusters have shifted toward mesenchymal stem cell‐derived exosomes, cell‐free therapeutic strategies, engineered exosomes, drug delivery systems, and regenerative medicine, reflecting a transition from mechanistic exploration to translational application. This shift has coincided with the growing prominence of journals such as the *International Journal of Molecular Sciences*, *Journal of Neurotrauma*, *Cells*, and *Neural Regeneration Research* as major publication venues for the field.

**Conclusion:**

This study maps the global landscape of exosomes research in TBI and traces its evolution from pathogenesis to regenerative therapy. The United States and China stand out as complementary leaders, with American groups focusing on mechanisms and biomarkers and Chinese groups driving therapeutic translation. Future priorities for clinical translation include standardized isolation protocols, optimal dosing regimens, and rigorous clinical trials. Together, these findings offer a practical framework to guide the development of exosome‐based therapies for TBI.

## Graphical abstract

A bibliometric map of exosome research in traumatic brain injury. Analysis of 609 publications from the Web of Science Core Collection and Scopus (2012‐2025; search completed October 24, 2025) maps the evolution of research emphasis from secondary injury and neuroinflammation toward stem‐cell‐derived exosomes, engineered vesicles, drug delivery and regenerative strategies. Priorities for clinical translation include standardized isolation, optimized dosing and rigorous clinical trials. Arrows denote the study workflow and evolution of research attention.

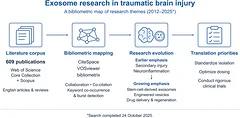

**Abbreviations**
AQP4: Aquaporin‐4
BBB: Blood–brain barrier
CDC: Centers for Disease Control and Prevention
CNS: Central nervous system
EVs: Extracellular vesicles
EXOs: Exosomes
IF: Impact factor
IL‐1β: Interleukin‐1 beta
IL‐6: Interleukin‐6
JCR: Journal Citation Reports
MCP: Multiple‐country publications
miRNAs: MicroRNAs
MSC: Mesenchymal stem cells
MSC‐Exos: Mesenchymal stem cell–derived exosomes
NIH: National Institutes of Health
SCP: Single‐country publications
SI: Secondary injury
TBI: Traumatic brain injury
TC: Total citations
TNF‐α: Tumor necrosis factor alpha
WOS: Web of Science
WoSCC: Web of Science Core Collection

## 1 Introduction

TBI is a major public health problem, defined as an alteration in brain function caused by an external force (Menon et al. 2010), and commonly results from falls and motor vehicle crashes (Dewan et al. 2019), as well as sports‐related incidents (Maas et al. 2017). Epidemiological data indicate that TBI affects millions of people each year (GBD 2016 Traumatic Brain Injury and Spinal Cord Injury Collaborators 2019); the Centers for Disease Control and Prevention (CDC) has reported approximately 2.80 million TBI‐related emergency department visits, hospitalizations, and deaths in the United States in 2013 (Taylor et al. 2017). The incidence is particularly high among young children, adolescents, and older adults, underscoring the need for targeted prevention and more effective therapies (Taylor et al. 2017; Maas et al. 2017).

Exosomes contribute to the pathophysiology of TBI by mediating intercellular communication and transporting bioactive molecules involved in neurorepair processes (Cai et al. 2025; Hu et al. 2025). Exosomes derived from bone marrow stem cells can enhance neurorepair after TBI by promoting angiogenesis and improving neurological function (Hu et al. 2025). Because they can cross the blood–brain barrier and deliver functional cargo, exosomes are promising agents for neuroregeneration, although standardization and safety remain challenges for clinical translation (Cai et al. 2025). Moreover, exosomes carry disease‐associated molecular cargo that can be detected in peripheral biofluids, supporting their development as minimally invasive biomarkers for TBI diagnosis and monitoring (Wang et al. 2018). Exosomes released from microglia and other cellular sources may further promote neuroprotection by modulating neuroinflammation and delivering neurotrophic and anti‐inflammatory mediators, thereby enhancing neuronal survival and functional recovery after TBI (Yang et al. 2026; Gong et al. 2025). Collectively, these findings underscore the pivotal role of exosomes in advancing diagnostic and therapeutic strategies for TBI by targeting neuroinflammation and facilitating neurorepair (Kabatas et al. 2025; Li et al. 2025).

Despite rapid progress, important gaps remain in our understanding of the mechanisms by which Exosomes regulate neuroinflammation and neurorepair in TBI, as well as their translational potential. The primary objective of this study was to evaluate the evolution of exosomes research in TBI from 2012 to 2025 using bibliometric and visual analyses. We performed co‐citation network analysis, keyword co‐occurrence analysis, and citation‐burst detection to identify the intellectual structure of the field, pivotal studies, and emerging trends. We also mapped the global research landscape by quantifying contributions from countries, institutions, authors, and journals to elucidate collaboration patterns and research productivity. By identifying current hotspots, gaps, and translational opportunities, this study provides a structured reference framework to inform future basic research, clinical translation, and policy development related to exosome‐based strategies in TBI.

## 2 Methods

### 2.1 Data Source and Search Strategy

All bibliometric data were retrieved on October 24, 2025 from the Web of Science Core Collection (WoSCC) and Scopus. Both databases were searched with an equivalent strategy combining traumatic brain injury and exosome terms: in WoSCC, TS = (“traumatic brain injury” [MeSH]) AND TS = (exosome OR “extracellular vesicle” OR exosomal); in Scopus, TITLE‐ABS‐KEY (“traumatic brain injury” [MeSH]) AND (exosome OR “extracellular vesicle” OR exosomal). Eligible records were English‐language original research articles or review articles published between January 1, 2012 and October 24, 2025; editorials, letters to the editor, conference abstracts, meeting proceedings, book chapters, and non‐English publications were excluded. Records from both databases were merged and deduplicated using R (v4.4.3) and Microsoft Excel, and after systematic screening 609 publications were retained for analysis (Figure S1).

### 2.2 Data Analysis

Scopus records were first converted into the Web of Science plain‐text format using CiteSpace, and the merged dataset was then analyzed with three complementary tools. CiteSpace (v6.4.R1, Drexel University) was used for co‐occurrence analysis, cluster detection, citation‐burst identification, and timeline visualization of reference clusters. VOSviewer (v1.6.18, Leiden University) was used to construct and visualize collaboration networks (country, institution, and author levels) and keyword co‐occurrence networks. The Bibliometrix R package (v4.4.3) was used for descriptive statistics (annual growth rate, citation metrics), productivity analysis (publication counts, h‐index, g‐index, m‐index), and relational network analysis (co‐citation, bibliographic coupling). Journal impact factors and quartile rankings (Q1–Q4) were obtained from the 2024 Journal Citation Reports (JCR), and Microsoft Excel was used for supplementary charts. The complete screening and analysis workflow is shown in Figure S1.

## 3 Results

### 3.1 Annual Publications Trends

The dataset comprised 609 documents published between 2012 and 2025. Annual output on TBI and exosomes rose steadily across this period, with marked acceleration after 2017 (Figure 1A). Because records were retrieved on October 24, 2025, the 2025 count may be incomplete; by that date, cumulative publications had reached 609 (Figure 1A). Original research articles comprised 79.8% of the corpus (*n* = 486) and review articles 20.2% (*n* = 123) (Table S1).

**FIGURE 1:**
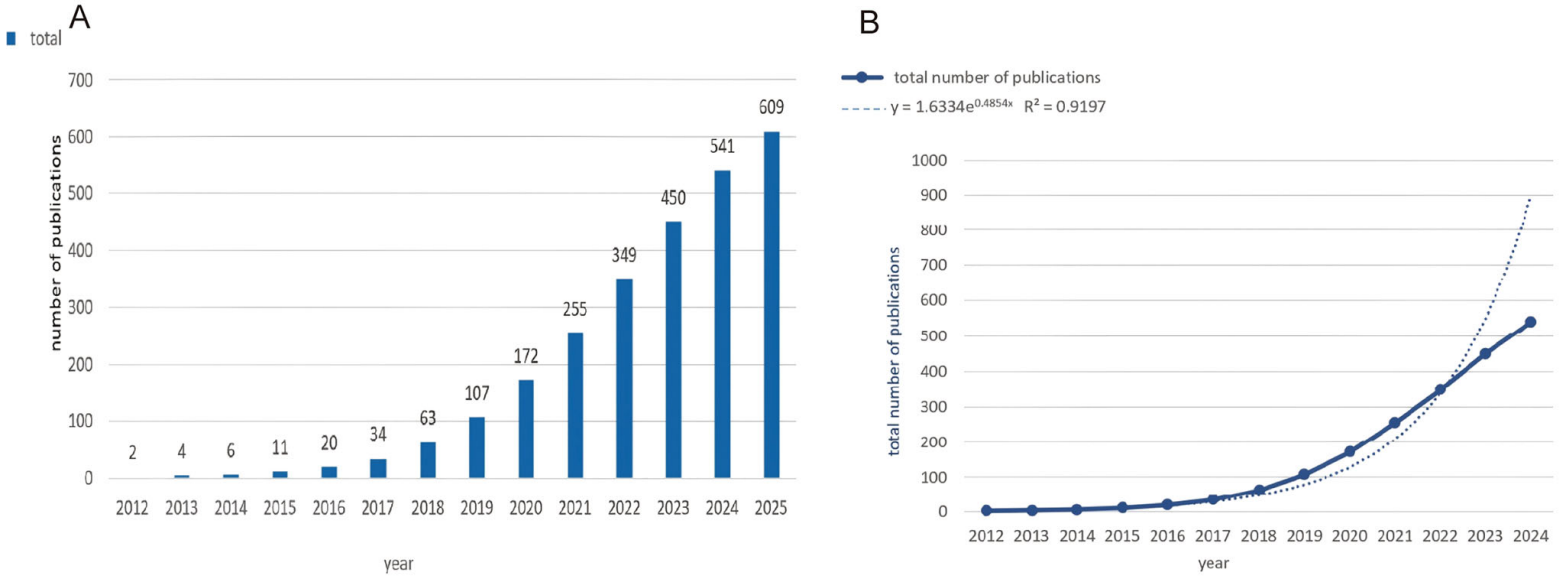
**Publication trends in TBI and EXOs research, 2012–2025**. (A) Cumulative number of publications over time. (B) Exponential growth model fitted to annual publication output (*y* = 1.6334e^0.4854^ *^x^*; *R* ^2^ = 0.9197). Data retrieved from WoSCC and Scopus on October 24, 2025.

An exponential growth model fitted to the publication data showed strong correlation (*R* ^2^ = 0.9197), confirming rapidly accelerating research activity (Figure 1B). The mean annual growth rate was 31.16%, with peak productivity in 2024 (Table S1). The corpus also demonstrated substantial scholarly impact (mean, 53.42 citations per document), drew on a large reference base (23,281 cited references), and remained relatively recent (mean document age, 3.31 years). Authorship patterns reflected strong collaborative engagement: 3084 authors contributed to the field, with only 7 single‐author publications and 12.97% of papers involving international co‐authorship (Table S1).

### 3.2 Country‐Level Research Output and International Collaboration

Research output was concentrated in China, the United States, and several European countries (Figure 2A). China (140 publications, 23.0%) and the United States (129 publications, 21.2%) were the most productive countries, together accounting for nearly half of all output (Table S2). Both nations produced predominantly single‐country publications (China: 114; United States: 107) while also contributing substantial multi‐country work, indicating a balance between domestic capacity and international collaboration. European countries—including Italy, Germany, the United Kingdom, Spain, France, and Croatia—contributed smaller but notable shares. The United Kingdom (83.3%) and France (100%) showed high proportions of multi‐country publications, whereas Germany and Spain had lower MCP ratios, reflecting a stronger domestic research orientation.

**FIGURE 2:**
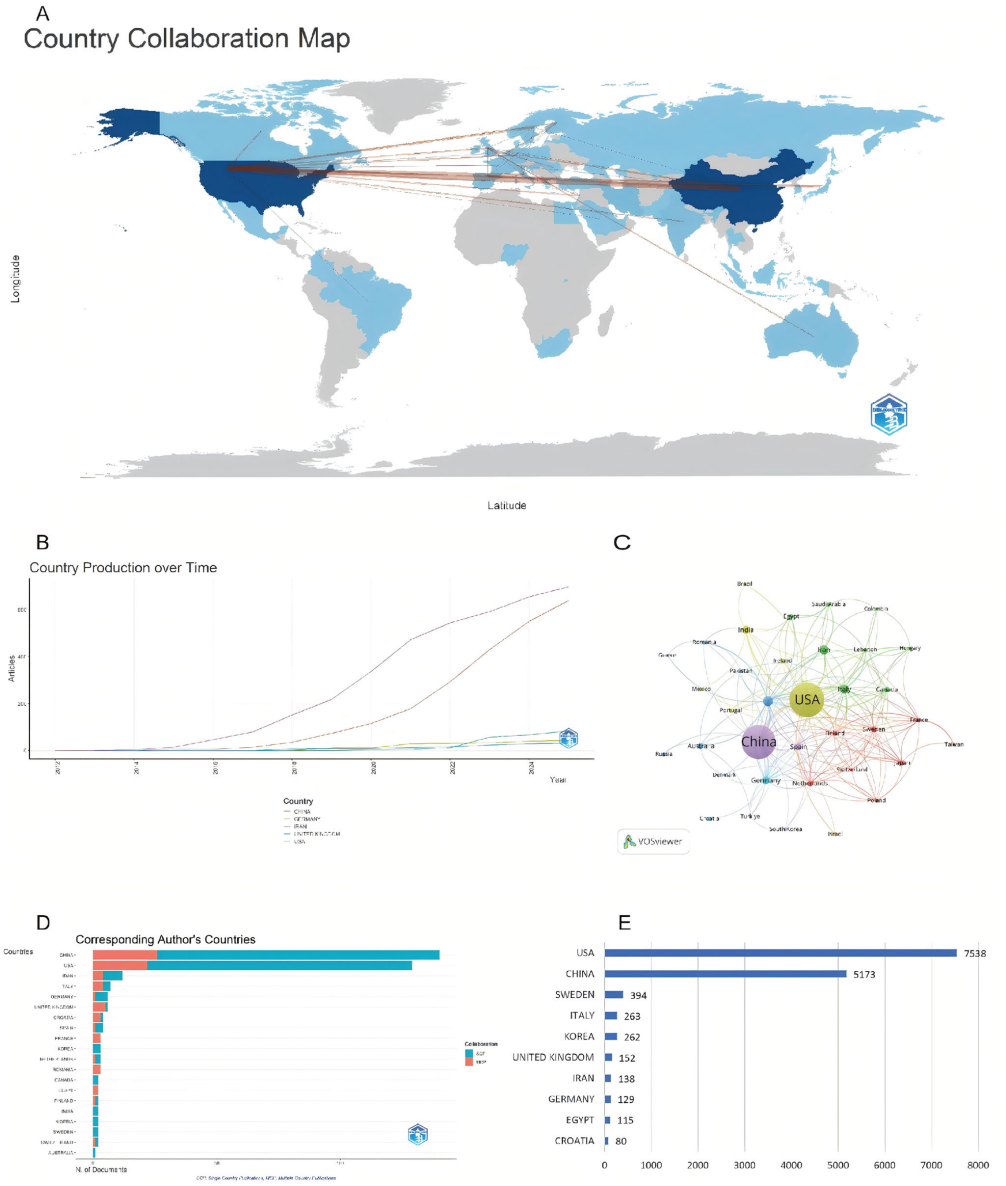
**Country‐level contributions and collaboration patterns in TBI and EXOs research**. (A) Global collaboration map. (B) Annual publication trends of the top 10 countries. (C) Country collaboration network generated in VOSviewer; node size = publication count, link thickness = collaboration strength. (D) Distribution of publications by corresponding author's country. (E) Top 10 countries by total citations (TC). Data from WoSCC and Scopus.

The United States led in citation impact with 7538 total citations and an average of 58.4 per article; Korea, despite only three publications, achieved the highest average citations (87.3). Figure 2B–E depicts the structural and temporal dimensions of the collaboration network. The United States, China, and several European nations occupied highly central positions, serving as key bridges in global cooperation. Temporal trends revealed heterogeneous growth trajectories across countries, reflecting the gradual expansion and diversification of research participation.

### 3.3 Authors Contributions in TBI and Exosomes Research

A total of 3084 authors contributed to the 609 publications between 2012 and 2025, yielding an average of 7.22 authors per document; single‐author publications were rare (*n* = 7), underscoring the dominance of team‐based research (Table S1). Table S3 ranks the most influential authors by productivity and citation impact. Chopp M led with 16 publications, 2199 citations, and the highest h‐index (15) since entering the field in 2015. Li Y and Xiong Y followed with 22 and 14 articles, respectively, both showing strong citation performance. Other prominent contributors included Zhang JN, Kapogiannis D, Zhang Y, Devoto C, Diaz‐Arrastia R, Dong JF, and Kenney K. Most of these researchers began publishing in this area after 2018, pointing to a recent influx of influential scholars.

The author collaboration network revealed a densely connected community, with several core authors maintaining extensive collaborative ties (Figure 3A). Multiple interconnected clusters suggest stable cooperation patterns and sustained scholarly interaction. In contrast, the co‐cited author network displayed a more modular structure, with well‐defined clusters representing the intellectual foundations of TBI‐exosome research (Figure 3B). Highly co‐cited authors were concentrated within a limited number of clusters, showing that much of the field draws on a relatively small group of foundational scholars.

**FIGURE 3:**
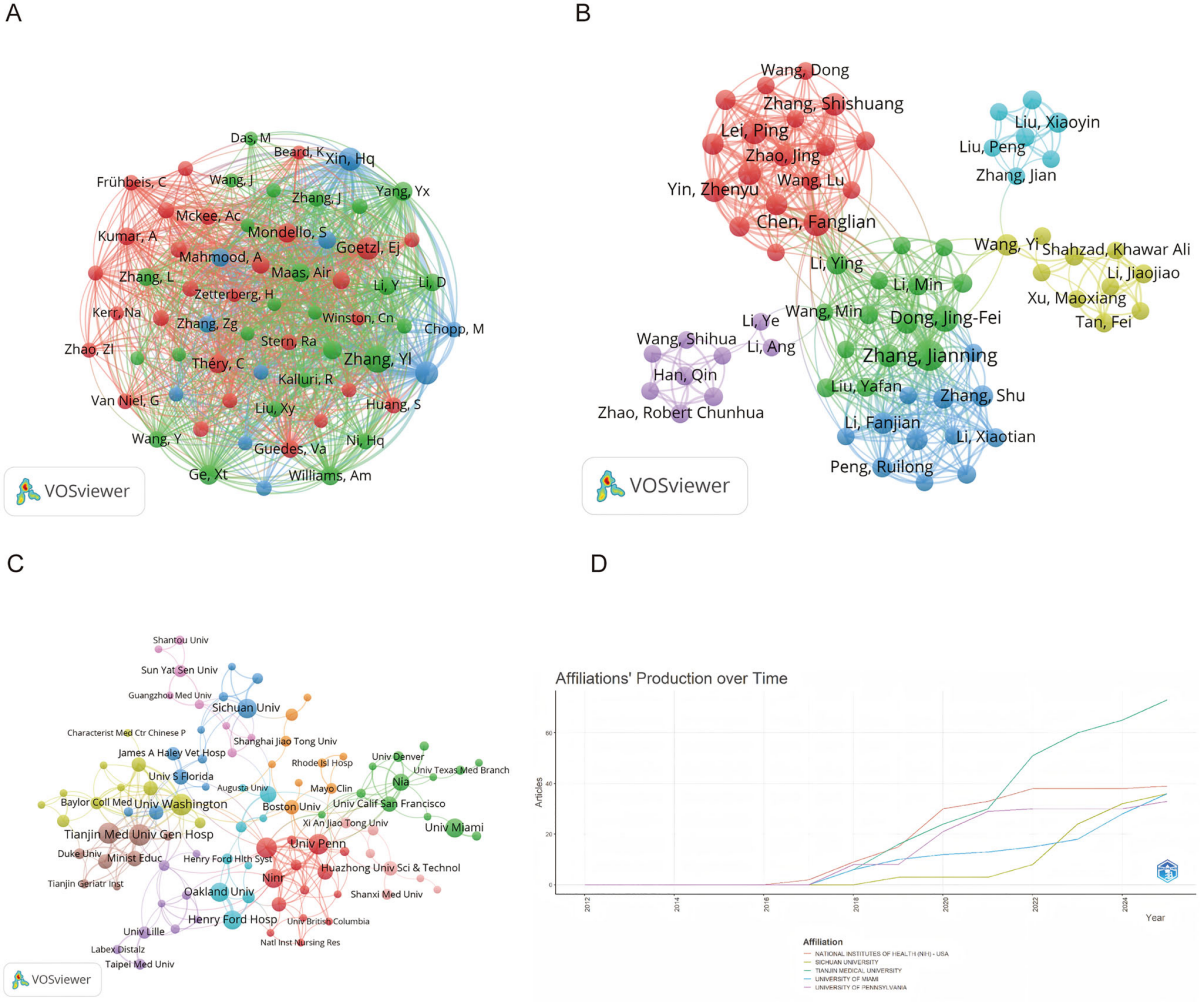
**Author and institutional contributions to TBI and EXOs research**. (A) Author collaboration network (VOSviewer); node size = publications, links = co‐authorship, colors = clusters. (B) Co‐citation network of cited authors; node size = citation frequency, links = co‐citation relationships. (C) Institutional collaboration network (VOSviewer); node size = publications, links = inter‐institutional collaboration. (D) Temporal trends in annual publication output of the top 10 institutions, 2012–2025.

### 3.4 Institutional Contributions in TBI and Exosomes Research

Major institutions in the United States and China occupied central positions in the collaboration network, with several medical universities and research hospitals serving as key hubs (Figure 3C). Consistent with this pattern, productivity rose steadily among leading organizations—including the National Institutes of Health, Sichuan University, Tianjin Medical University, the University of Miami, and the University of Pennsylvania—particularly after 2018 (Figure 3D). These observations indicate that a relatively small group of institutions has played a dominant role in sustaining and expanding research activity in this field.

### 3.5 Journals and Source Impact Analysis

Table S4 lists the 15 most influential journals in this field. The “*International Journal of Molecular Sciences*” ranked first (h‐index = 17, g‐index = 29, TC = 1053, NP = 29), underscoring its central role in disseminating molecular studies of exosomes in TBI. The “*Journal of Neurotrauma”* ranked second (h‐index = 16, TC = 837), reflecting its prominence as a specialty journal in neurotrauma research. Other influential journals, including “*Cells,”* “*Neural Regeneration Research,”* and “*Frontiers in Cellular Neuroscience,”* showed balanced productivity and citation impact, pointing to increasing interdisciplinary integration across neuroscience, cell biology, and regenerative medicine.

Bradford's Law identified a core journal group contributing a disproportionate share of publications (Figure 4B). Source co‐citation and bibliographic coupling analyses revealed a highly interconnected journal network (Figure 4C, D), with the “*Journal of Neurotrauma*,” “*Cells,”* and the “*International Journal of Molecular Sciences”* occupying central positions that link basic molecular research with clinical neurotrauma studies. Temporal analysis showed marked growth in journal output after 2018, with accelerated increases from 2020 onward (Figure 4E), likely reflecting advances in exosome biology and translational neuroscience.

**FIGURE 4:**
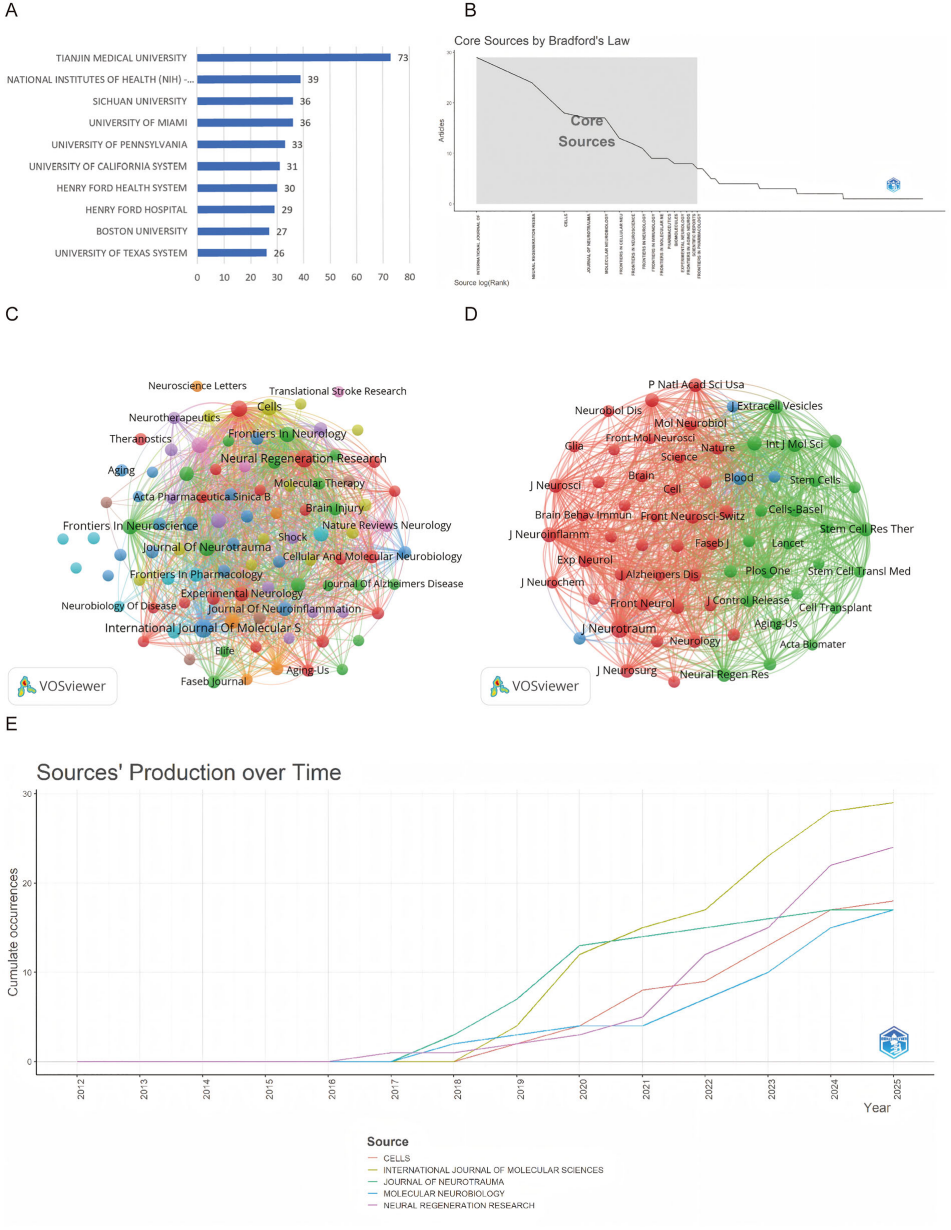
**Journal distribution and citation characteristics in TBI and EXOs research**. (A) Top 10 most productive journals ranked by publication count. (B) Bradford's Law analysis identifying core journal sources; shaded zone = core journals. (C) Journal co‐occurrence network; node size = article count, links = co‐occurrence, colors = clusters. (D) Journal co‐citation network; node size = citations, links = co‐citation. (E) Cumulative publication trends of major journals, 2012–2025.

### 3.6 Articles and References Analysis

Table S5 summarizes the 10 most cited publications. The most frequently cited was Kalluri and LeBleu (2020), “The biology, function, and biomedical applications of exosomes,” published in “*Science*” in 2020 (7821 total citations; 1303.50 per year; normalized total citations = 42.99), establishing its foundational influence in exosome biology. Several highly cited studies addressed therapeutic applications: Zhang YL (2015) showed that mesenchymal stromal cell‐derived exosomes promoted functional recovery and neurovascular plasticity in a rat TBI model (578 citations) (Zhang et al. 2015), and Zhang YL (2017) reported that bone marrow‐derived mesenchymal stem cell exosomes improved neurological outcomes after TBI (314 citations)(Zhang et al. 2017). Influential reviews included Zhang ZG (2019) on exosomes as restorative therapies for neurological injury (“*Nature Reviews Neurology*,” 432 citations) (Zhang et al. 2019), and Wang KKW (2018) on diagnostic and prognostic biomarkers for TBI (“*Expert Review of Molecular Diagnostics*,” 422 citations) (Wang et al. 2018). Recent literature has emphasized clinical translation: Tan F (2024) reviewed clinical applications of stem cell‐derived exosomes in “*Signal Transduction and Targeted Therapy*,” achieving the highest citations per year (182.00) among the top 10 articles (Tan et al. 2024), and Gupta D (2021) discussed exosome dosing strategies in “*Advanced Drug Delivery Reviews*” (Gupta et al. 2021).

Co‐citation analysis revealed a well‐organized intellectual structure underlying TBI‐exosome research (Figure 5A, B). The network comprised 682 nodes and 1184 links (density = 0.0054) and could be partitioned into multiple well‐defined clusters centered on TBI pathology, extracellular vesicle biology, and exosome‐related mechanisms, forming the core knowledge base of the field. A timeline visualization of co‐cited reference clusters from 2012 to 2025 (Figure 5C) showed an evolution from early work on acute brain injury mechanisms, neuroinflammation, and fundamental exosome characterization toward more prominent clusters involving exosomal microRNAs, chronic traumatic encephalopathy, and neuroprotective effects. The most recent period saw continued activity in clusters related to MSC‐derived exosomes, vesicular drug delivery systems, and engineered extracellular vesicles, reflecting an increasing emphasis on translational research.

**FIGURE 5:**
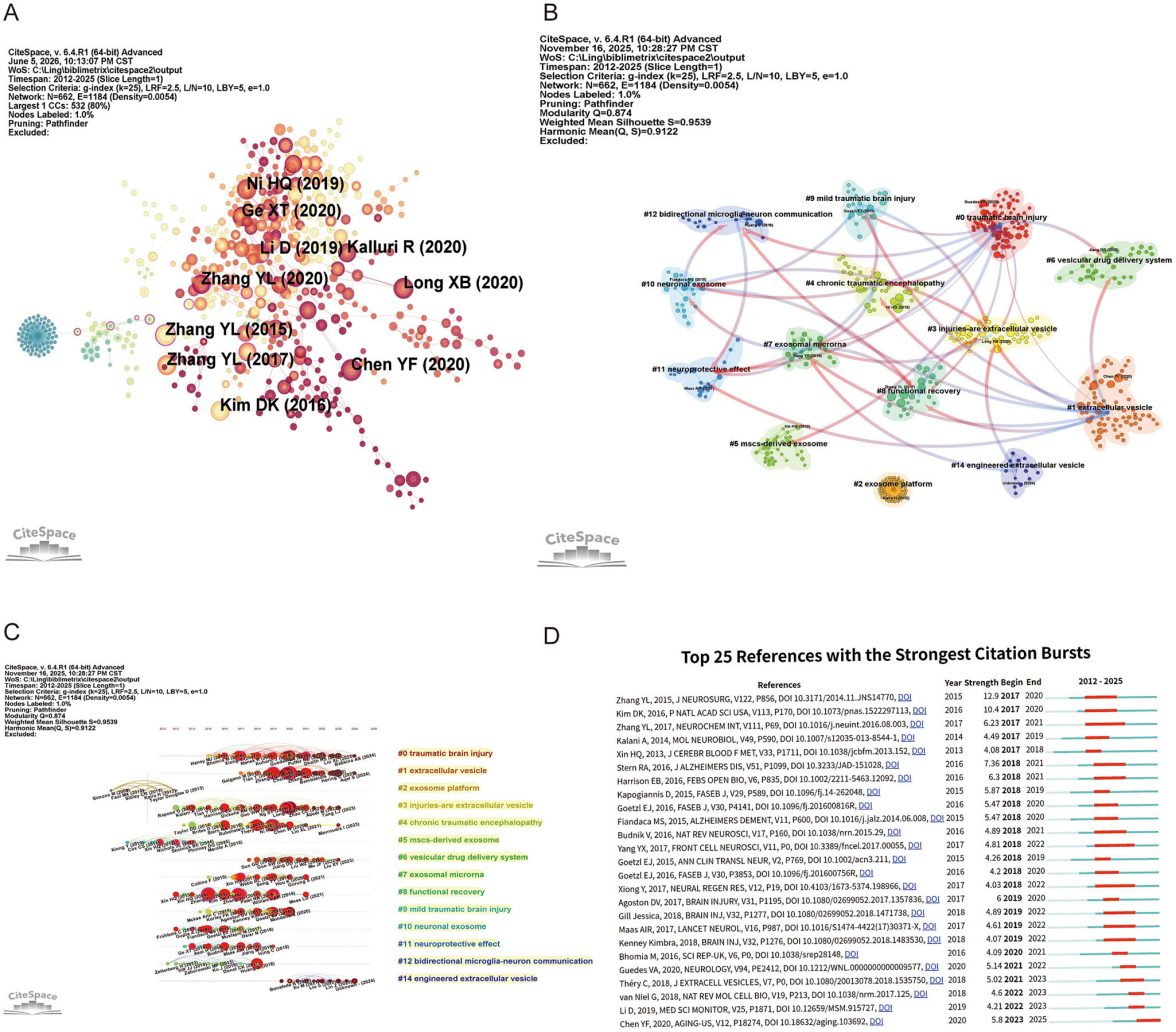
**Reference co‐citation analysis in TBI and EXOs research**. (A) Co‐citation network (CiteSpace); node size = citations, links = co‐citation, node color = publication year. (B) Clustered co‐citation network; colors denote thematic clusters. (C) Timeline view of reference clusters; each line = one cluster, red rings = citation bursts. (D) Top 25 references with the strongest citation bursts, 2012–2025. Strength = burst intensity; Begin/End = burst duration; red bars = active burst periods.

Citation‐burst analysis of the top 25 references (Figure 5D) showed that earlier burst references (2017–2020) primarily represented foundational work on exosome characterization, neuroinflammation, and central nervous system injury. By contrast, the most recent burst references (2023–2025) signaled growing attention to neural stem cell‐related regenerative strategies, the interface between TBI and neurodegenerative diseases, and broader brain disease contexts.

### 3.7 Keyword Analysis

The keyword network was highly interconnected, with “traumatic brain injury,” “extracellular vesicles,” and “exosomes” occupying central positions (Figure 6A), indicating that extracellular vesicles have become a core framework for mechanistic and translational investigations in TBI research. Terms related to neuroinflammation, microglia, the blood–brain barrier, and secondary injury formed tightly connected clusters (Figure 6B), reflecting sustained interest in post‐traumatic pathophysiology, while clusters associated with mesenchymal stem cells, neuroprotection, cell‐free therapy, and drug delivery point to growing emphasis on extracellular vesicle‐based therapeutics. Keyword frequency analysis (Figure 6C) confirmed these patterns, with the top terms followed by neuroinflammation, microglia, and biomarkers. Figure 6D further showed frequent co‐occurrence of human, nonhuman, and animal models alongside the term “review,” reflecting parallel experimental and clinical research tracks. Keywords related to neurodegenerative diseases, including Alzheimer's and Parkinson's disease, also suggest increasing attention to the long‐term neurological consequences of TBI.

**FIGURE 6:**
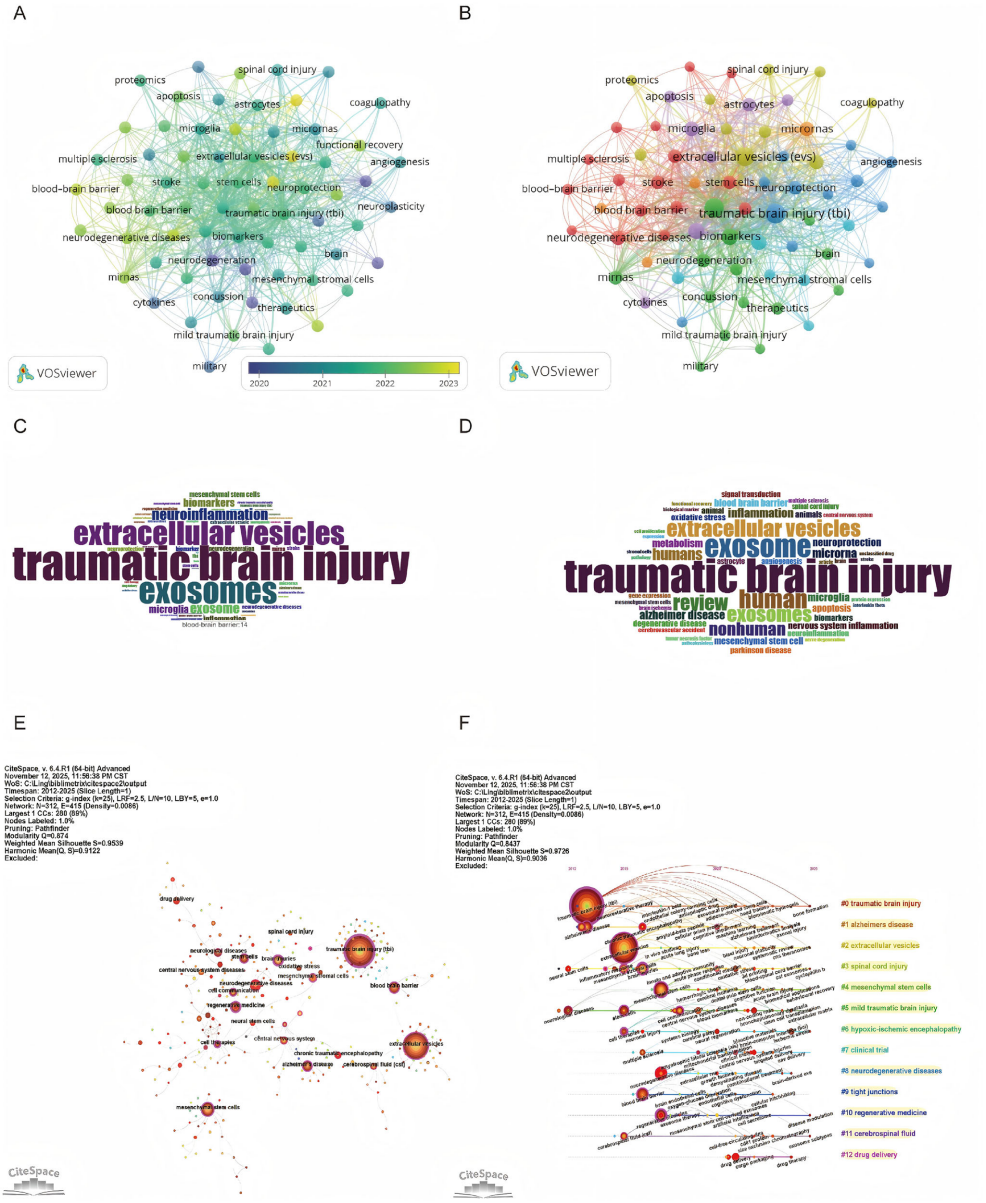
**Keyword analysis in TBI and EXOs research**. (A) Keyword co‐occurrence overlay network (VOSviewer); node size = frequency, node color = mean publication year (blue = earlier, yellow = more recent). (B) Clustered keyword network showing thematic groups. (C) Word cloud of author keywords; font size = frequency. (D) Word cloud of Keywords Plus. (E) Keyword burst network (CiteSpace); larger nodes with red rings = strong citation bursts. (F) Timeline of keyword clusters; red segments = burst periods.

Keyword burst detection at the network level (Figure 6E) showed that early burst keywords centered on the blood–brain barrier, glial injury, and secondary injury, consistent with foundational mechanistic studies. Terms related to mesenchymal stem cells, regenerative medicine, and cell‐free therapy subsequently emerged, signaling a shift toward therapeutic development. The timeline view of keyword clusters (Figure 6F) demonstrated a parallel evolution: early studies focused on acute injury mechanisms and central nervous system pathology, whereas recent research has emphasized extracellular vesicle‐based delivery systems, regenerative medicine, and functional recovery.

The top 25 keywords with citation bursts (Figure 7) further refined the identification of research frontiers. Early bursts (2014–2018), including “inflammatory response,” “secondary injury,” and “cerebrospinal fluid,” reflected an initial focus on acute injury responses and diagnostic indicators. Between 2016 and 2020, mechanistically oriented bursts such as “neuronal injury,” “hypoxic‐ischemic encephalopathy,” “microtubule‐associated protein tau,” and “glymphatic system” highlighted growing interest in neuronal degeneration and clearance pathways. In the most recent period (2021–2025), burst keywords shifted toward “drug delivery,” “non‐coding RNAs,” “adipose‐derived mesenchymal stem cells,” “neural stem cells,” and “neurodegenerative diseases,” marking an increasingly translational and long‐term disease‐oriented focus.

**FIGURE 7:**
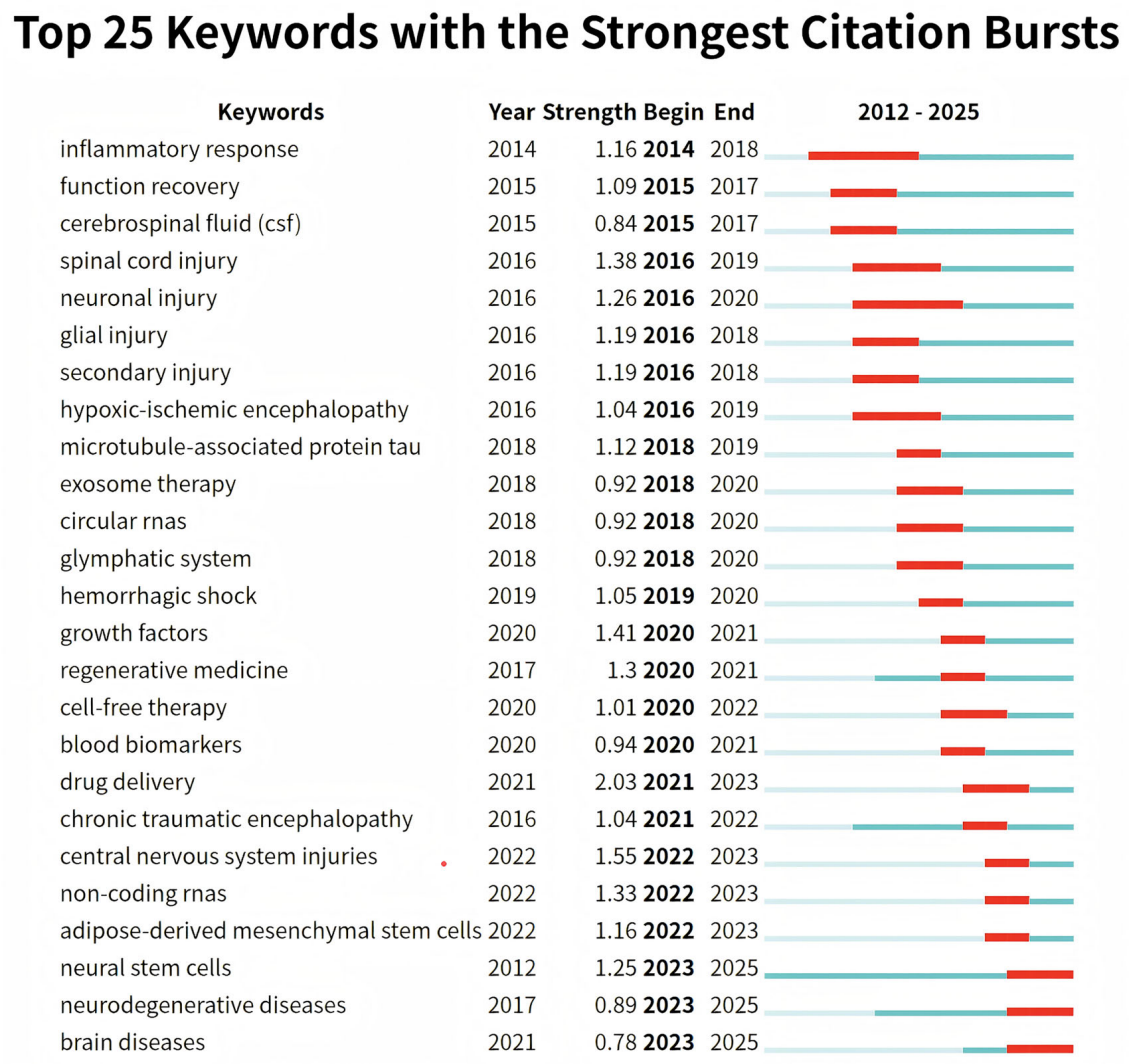
**Top 25 keywords with the strongest citation bursts in TBI and EXOs research, 2012–2025**. Each row = one keyword. Strength = burst intensity; Begin/End = burst interval. Red bars = active burst periods. Blue line = study period boundary (2025 data retrieved October 24, 2025; may be incomplete).

## 4 Discussion

This bibliometric analysis of 609 publications shows that research on exosomes in TBI has grown exponentially since 2017 (*R* ^2^ = 0.9197; mean annual growth rate, 31.16%). The United States and China dominate the field with complementary profiles: American groups lead in citation impact and mechanistic/biomarker research, whereas Chinese groups lead in publication volume and therapeutic translation. Keyword‐burst and co‐citation analyses reveal a clear thematic evolution, from early bursts centered on secondary‐injury mechanisms (2014–2018: “inflammatory response,” “secondary injury,” “blood–brain barrier”) to recent bursts centered on translational themes (2021–2025: “drug delivery,” “non‐coding RNAs,” “neural stem cells,” “regenerative medicine”). The intellectual base of the field rests on a relatively small group of foundational scholars, most notably Chopp M and Zhang Y, whose highly co‐cited work established exosome‐mediated neuroprotection and neurovascular remodeling as core concepts. In the remainder of this Discussion, each theme is interpreted strictly through this bibliometric evidence, linking the underlying biology to the keyword bursts, co‐citation clusters, and citation analyses that document how and when the theme became prominent.

### 4.1 Secondary Injury and Neuroinflammation

Secondary injury and neuroinflammation constitute the founding theme of this field. The earliest keyword bursts (2014–2018) were “inflammatory response,” “secondary injury,” and “cerebrospinal fluid” (Figure 7), and the earliest burst references (2017–2020) addressed neuroinflammation and central nervous system injury (Figure 5D); neuroinflammation‐ and microglia‐related terms still form one of the most tightly connected keyword clusters (Figure 6B). Mechanistically, secondary injury is a delayed, progressive cascade driven by oxidative stress, excitotoxicity, and blood–brain barrier disruption (Sipos et al. 2026). Its defining feature is excessive release of interleukin‐1β (IL‐1β), interleukin‐6 (IL‐6), and tumor necrosis factor‐α (TNF‐α), which sustain microglial activation; persistently activated microglia amplify neuronal apoptosis through continued cytokine and reactive oxygen species production, establishing a self‐perpetuating inflammatory loop (Ho et al. 2026). In severe TBI, such secondary neuroinflammation can persist in remote regions such as the thalamus for months and is associated with neuronal loss and disturbed corticothalamic connectivity (Dvořáková et al. 2026). Blood–brain barrier breakdown further increases vascular permeability, permitting infiltration of peripheral immune mediators and intensifying neuroinflammation (Komiotis et al. 2026), while microglia‐derived extracellular vesicles can modulate resident central nervous system cells after acute brain injury, providing a direct rationale for exosome‐based anti‐inflammatory strategies (Gong et al. 2025). Notably, this cluster has remained continuously active from 2014 to the present—unlike short single‐year bursts elsewhere in the network—indicating that neuroinflammation is a durable, cross‐cutting research priority rather than a transient fashion.

### 4.2 Glial Interactions and Neuroimmune Regulation

“Microglia” is among the highest‐frequency keywords in the corpus and occupies a central position within the neuroinflammation keyword cluster (Figure 6B, C), and glial‐injury terms appear among the early burst keywords (Figure 6E), identifying glial biology as an early and persistent hotspot. Astrocytes and microglia coordinate post‐traumatic immune signaling through cytokine and chemokine release, shape blood–brain barrier integrity, and thereby regulate immune cell recruitment and persistence in the injured brain. Microglia respond dynamically to astrocytic cues and injury‐associated signals, adopting context‐dependent phenotypes ranging from neuroprotective to neurotoxic, and bidirectional microglia‐neuron communication is increasingly recognized as a determinant of outcome after brain injury (Szepesi et al. 2018). Studies in neurodegenerative disease have linked microglial activation states and microglia‐specific genetic risk to adverse neurological and cognitive outcomes (Patil et al. 2025; Mendez et al. 2025), and microglia‐derived extracellular vesicles can propagate or dampen inflammatory signaling among resident central nervous system cells (Gong et al. 2025). The sustained centrality of glial keywords therefore mirrors a mechanistic consensus that astrocyte‐microglia crosstalk is a critical driver of post‐traumatic neuroinflammation and a plausible target for exosome‐based intervention.

### 4.3 Exosomal Cargo and Molecular Regulation

Exosomal cargo emerged as a frontier only recently: “non‐coding RNAs” is among the 2021–2025 burst keywords (Figure 7), and the reference co‐citation timeline shows a prominent cluster on exosomal microRNAs (Figure 5C). Exosomal microRNAs repress target mRNAs through sequence‐specific binding and thereby modulate inflammatory responses, apoptosis, and cellular metabolism in recipient cells; microRNAs from human umbilical cord mesenchymal stem cell‐derived exosomes, for example, alter gene expression and cellular behavior in target cells (Khezrian et al. 2025). In TBI models, this cargo biology has been exploited therapeutically: an injectable hydrogel loaded with exosomes from miR‐124‐3p‐overexpressing microglia attenuated injury (Yang et al. 2026), and induced neural stem cell‐derived exosomes promoted recovery through microglial modulation (Li et al. 2025). The recent timing of these bursts and clusters indicates that cargo engineering is a young but rapidly consolidating research direction.

### 4.4 Blood–Brain Barrier and the Glymphatic System

Barrier and clearance biology spans the early and middle phases of the field: “blood–brain barrier” featured among the early burst keywords, whereas “glymphatic system” and “microtubule‐associated protein tau” burst between 2016 and 2020 (Figure 7), and the co‐citation timeline contains a chronic traumatic encephalopathy cluster (Figure 5C) linking clearance failure to long‐term TBI sequelae. Blood–brain barrier integrity is functionally coupled to glymphatic clearance: altered polarity of aquaporin‐4 channels along astrocytic end‐feet disturbs perivascular fluid transport and exacerbates neuroinflammation (Brehar et al. 2025). Structural cranial defects such as those created by craniectomy can themselves alter tight junction protein expression and increase blood–brain barrier permeability, accompanied by neuroinflammatory changes (Tarudji et al. 2025), while impaired glymphatic function limits amyloid‐βclearance and promotes cerebral amyloid angiopathy and long‐term neurodegenerative change (Zupan et al. 2026). Elevated intracranial pressure can further restrict cerebrospinal fluid influx and glymphatic clearance, a mechanism with potential implications for secondary injury (Xiang et al. 2022), and restoration of cranial structure by cranioplasty can partially normalize barrier function (Ojogho et al. 2025). The sequential timing of these bursts—barrier first, clearance and tau later—tracks the field's shift from acute pathology toward the chronic consequences of TBI.

### 4.5 Cell‐Free Therapy and Regenerative Medicine

Cell‐free therapy is the dominant current frontier: the most recent keyword bursts (2021–2025) include “drug delivery,” “adipose‐derived mesenchymal stem cells,” “neural stem cells,” and “regenerative medicine” (Figure 7), and the most recent co‐citation clusters concern mesenchymal stem cell (MSC)‐derived exosomes, engineered extracellular vesicles, and vesicular drug delivery systems (Figure 5C). Three of the ten most cited publications in the corpus are MSC‐exosome TBI studies: systemic administration of MSC‐derived exosomes improved functional recovery and neurovascular plasticity after TBI in rats (Zhang et al. 2015), with subsequent work demonstrating that exosomes generated by human bone marrow MSCs cultured under both 2D and 3D conditions improve functional recovery after TBI, informing production strategies for cell‐free exosome therapy (Zhang et al. 2017), and chromatographically isolated CD63+CD81+ extracellular vesicles from mesenchymal stromal cells rescued cognitive impairments after TBI (Kim et al. 2016). More recent preclinical studies report that MSC‐derived exosomes attenuate neurodegeneration, preserve cortical architecture, and improve cognition in repetitive TBI models (Refat M. Selim et al. 2025), with emerging efficacy and safety data for Wharton's jelly‐derived MSC exosomes (Kabatas et al. 2025) and biomaterial‐assisted delivery via injectable hydrogels (Yang et al. 2026). At the same time, a recent critical reappraisal urges more rigorous efficacy metrics and modeling standards for MSC‐extracellular vesicle therapies in acute brain injury (Zhou et al. 2025), indicating that this frontier is maturing from enthusiasm toward methodological consolidation.

### 4.6 Comparative Analysis of National Research Priorities and Translational Challenges

Our bibliometric analysis identified China (23.0%) and the United States (21.2%) as the dominant contributors to TBI‐exosomes research (Table S2). Chinese research has focused on therapeutic translation, with studies demonstrating that stem cell‐derived exosomes promote angiogenesis, modulate microglial responses, and improve functional recovery in TBI models (Hu et al. 2025; Yang et al. 2026; Li et al. 2025). In contrast, US research has emphasized mechanistic understanding and biomarker development, establishing frameworks for diagnostic and prognostic biomarkers (Wang et al. 2018) and pioneering MSC‐derived exosome therapy in TBI models (Zhang et al. 2015; Zhang et al. 2017). European groups have contributed mechanistic insight into microglia‐neuron communication (Szepesi et al. 2018), and efficacy and safety data are also emerging from the Middle East (Kabatas et al. 2025; Refat M. Selim et al. 2025). These complementary strengths underscore the value of sustained international collaboration (Figure 3C).

Several factors plausibly explain this bipolar structure, and we offer them here as interpretation of the bibliometric patterns rather than new data. The United States entered the field earlier—its leading authors have been active since 2015 (Table S3)—and is supported by a mature National Institutes of Health and Department of Defense funding ecosystem for neurotrauma, which favors the mechanism‐ and biomarker‐oriented research consistent with its citation leadership (7538 total citations; 58.4 per article). China's rapid post‐2018 expansion coincides with national programs prioritizing stem‐cell and regenerative‐medicine research, yielding volume leadership (140 publications) but lower average citations (37.0 per article), a profile typical of younger, translation‐oriented output. Notably, the international co‐authorship rate is low (12.97%), and US–China co‐publication remains limited relative to the two countries’ combined output. We interpret this gap as a missed synergy: bilateral consortia combining American mechanistic depth with Chinese translational scale would likely accelerate clinical translation of exosome‐based therapies.

### 4.7 Emerging Themes and Translational Priorities

The bibliometric signals allow the emerging themes to be ranked by translational readiness. First, MSC‐derived exosome therapy is the most mature direction: it combines sustained multi‐year keyword bursts, a dense co‐citation cluster, established efficacy across different production conditions (2D vs. 3D culture) (Zhang et al. 2017), and emerging safety data (Kabatas et al. 2025), although concerns about efficacy metrics remain (Zhou et al. 2025); it is therefore the most likely theme to reach clinical trials first. Second, exosome biomarkers are nearest to routine clinical use: an established diagnostic and prognostic framework (Wang et al. 2018) and the prominence of biomarker‐focused investigators such as Kapogiannis D among the field's most influential authors, together with a lower regulatory barrier than for therapeutics, favor early adoption. Third, engineered exosomes and drug delivery show the strongest recent burst activity but the youngest clusters; manufacturing, dosing, and biodistribution hurdles (Gupta et al. 2021; Tan et al. 2024) imply transformative potential over a longer horizon. Fourth, the neurodegenerative‐disease linkage (chronic traumatic encephalopathy, tau) shows a sustained mid‐period burst and is likely durable because the TBI‐neurodegeneration connection is biologically grounded rather than a passing trend.

Not every burst, however, signals a durable priority. Short single‐year bursts associated with small clusters may reflect publication fashion, whereas themes that combine multi‐year bursts, growing co‐citation mass, and cross‐country adoption—criteria best met by MSC‐exosome therapy and biomarker research—are more likely to prove transformative. On this basis, we identify three practical priorities for the field: standardized isolation and characterization of exosome preparations (Kalluri and LeBleu 2020; Hade et al. 2021); rigorous dosing and biodistribution studies (Gupta et al. 2021); and GMP‐compliant scale‐up coupled with well‐designed clinical trials (Tan et al. 2024).

### 4.8 Limitations

This study provides the first comprehensive bibliometric overview of exosome research in TBI, but several limitations should be noted. First, the analysis relied on publications indexed in the WoSCC and Scopus and was restricted to English‐language literature; relevant studies in other databases or languages may therefore have been missed, introducing database and language bias. Second, bibliometric indicators such as citation counts, co‐citation networks, and keyword bursts reflect academic attention and knowledge dissemination rather than experimental rigor or biological validity. Highly cited studies and influential clusters therefore indicate intellectual impact rather than definitive mechanistic or clinical evidence, and recently published work may be underrepresented because citations take time to accumulate. Third, minor discrepancies among clustering and network structures may arise from methodological differences among CiteSpace, VOSviewer, and Bibliometrix; cluster boundaries and thematic labels should thus be interpreted as heuristic rather than absolute classifications. Finally, bibliometric analysis cannot directly address key translational challenges in exosome‐based therapies, including dosing, biodistribution, standardization, and safety. Accordingly, the trends identified here should be viewed as a conceptual framework that complements (rather than replaces) mechanistic studies and clinical investigations.

## 5 Conclusion

Our research found that themes have shifted from secondary‐injury mechanisms (neuroinflammation, microglial activation, blood–brain barrier disruption) toward mesenchymal stem cell‐derived exosomes, engineered vesicles, drug delivery, and regenerative medicine. These patterns imply that clinical translation now depends on standardized isolation and dosing protocols, GMP‐compliant production, rigorous clinical trials, and deeper cross‐national collaboration.

## Author Contributions

All authors contributed equally. All authors have read and agreed to the published version of the manuscript.

## Funding

The authors have nothing to report.

## Ethics Statement

This study is a bibliometric analysis based solely on publicly available literature data retrieved from the Web of Science Core Collection and Scopus databases. No human participants, animals, or identifiable personal data were involved in this research. Therefore, ethical approval and informed consent were not required.

## Conflicts of Interest

There are no disclosed conflicts of interest for the authors.

## Supporting information




**Supplementary Material**: brb371897‐sup‐0001‐SuppMat.docx

## Data Availability

The original data that support the conclusion of this paper will be provided by the authors in the article and Supplementary Material. Further inquiries can be directed to the corresponding authors.
