# Supplementary material for: Exosomes in Traumatic Brain Injury: From Secondary Injury Mechanisms to Cell‐Free Neurorepair
Source: Brain Behav. 2026 Nov 3;16(11):e71897. doi: 10.1002/brb3.71897 (PMC13729824; doi:10.1002/brb3.71897)
Supplement: Supplementary file 1 [file BRB3-16-e71897-s001.docx]

**Figure S1. Flowchart for literature search, selection, and analysis. TI: title; AK: author keywords; AB: abstract.**

**Supplementary Tables**

**Supplementary Table 1.** Bibliometric Summary of TBI and EXOs-Related Publications

**Supplementary Table 2.** Most Relevant Countries by Corresponding Author Contributions

**Supplementary Table 3.** Top 10 Authors on TBI and EXOs Research

**Supplementary Table 4.** Top 15 Journals by Influence Rank According to Bibliometrix Analysis

**Supplementary Table 5.**Top 10 Most Cited Publications Based on Bibliometrix Analysis

**Supplementary Table 1.**Bibliometric Summary of TBI and EXOs-Related Publications

| **Description** | **Results** |
| --- | --- |
| Timespan | 2012:2025 |
| Sources (Journals, Books, etc) | 271 |
| Documents | 609 |
| Annual Growth Rate % | 31.16 |
| Document Average Age | 3.31 |
| Average citations per doc | 53.42 |
| References | 23281 |
| DOCUMENT CONTENTS |  |
| Keywords Plus (ID) | 5262 |
| Author's Keywords (DE) | 1283 |
| AUTHORS |  |
| Authors | 3084 |
| Authors of single-authored docs | 7 |
| AUTHORS COLLABORATION |  |
| Single-authored docs | 7 |
| Co-Authors per Doc | 7.22 |
| International co-authorships % | 12.97 |
| DOCUMENT TYPES |  |
| article | 486 |
| review | 123 |

**Supplementary Table 2.** Most Relevant Countries by Corresponding Author Contributions

| **Rank** | **Country** | **Articles** | **Articles %** | **SCP** | **MCP** | **MCP %** | **TC** | **Average Article Citations** |
| --- | --- | --- | --- | --- | --- | --- | --- | --- |
| 1 | CHINA | 140 | 23 | 114 | 26 | 18.6 | 5173 | 37.00 |
| 2 | USA | 129 | 21.2 | 107 | 22 | 17.1 | 7538 | 58.40 |
| 3 | IRAN | 12 | 2 | 8 | 4 | 33.3 | 138 | 11.50 |
| 4 | ITALY | 7 | 1.1 | 3 | 4 | 57.1 | 263 | 37.60 |
| 5 | GERMANY | 6 | 1 | 5 | 1 | 16.7 | 129 | 21.50 |
| 6 | UNITED KINGDOM | 6 | 1 | 1 | 5 | ss83.3 | 152 | 25.30 |
| 7 | CROATIA | 4 | 0.7 | 1 | 3 | 75 | 80 | 20.00 |
| 8 | SPAIN | 4 | 0.7 | 3 | 1 | 25 | 70 | 17.50 |
| 9 | FRANCE | 3 | 0.5 | 0 | 3 | 100 | 30 | 10.00 |
| 10 | KOREA | 3 | 0.5 | 3 | 0 | 0 | 262 | 87.30 |

Abbreviation: SCP :Single Country Publications; MCP :Multiple Country Publications; TC: Total Citation

**Supplementary Table 3.**Top 10 Authors on TBI and EXOs Research

| **Rank** | **Author** | **h_index** | **g_index** | **m_index** | **TC** | **NP** | **PY_start** | **Articles** | **Articles Fractionalized** |
| --- | --- | --- | --- | --- | --- | --- | --- | --- | --- |
| 1 | CHOPP M | 15 | 16 | 1.364 | 2199 | 16 | 2015 | 16 | 3.78 |
| 2 | LI Y | 13 | 22 | 1.182 | 1260 | 22 | 2015 | 22 | 2.72 |
| 3 | XIONG Y | 12 | 14 | 1.091 | 1624 | 14 | 2015 | 14 | 2.45 |
| 4 | ZHANG JN | 12 | 17 | 1.5 | 824 | 17 | 2018 | 17 | 1.65 |
| 5 | KAPOGIANNIS D | 11 | 12 | 1.375 | 568 | 12 | 2018 | 12 | 1.99 |
| 6 | ZHANG Y | 11 | 18 | 1.571 | 511 | 18 | 2019 | 18 | 2.64 |
| 7 | DEVOTO C | 10 | 10 | 1.25 | 401 | 10 | 2018 | 10 | 0.82 |
| 8 | DIAZ-ARRASTIA R | 10 | 11 | 1.25 | 500 | 11 | 2018 | 11 | 1.19 |
| 9 | DONG JF | 10 | 11 | 1.25 | 273 | 11 | 2018 | 11 | 1.67 |
| 10 | KENNEY K | 10 | 11 | 1.25 | 454 | 11 | 2018 | 11 | 1.19 |

**Supplementary Table 4. Top 15 Journals by Influence Rank According to Bibliometrix Analysis**

| **Rank** | **Source** | **h_index** | **g_index** | **m_index** | **TC** | **NP** | **PY_start** | **Articles** | **JCR** | **IF** |
| --- | --- | --- | --- | --- | --- | --- | --- | --- | --- | --- |
| **1** | INTERNATIONAL JOURNAL OF MOLECULAR SCIENCES | 17 | 29 | 2.429 | 1053 | 29 | 2019 | 29 | Q3 | 4.9 |
| **2** | JOURNAL OF NEUROTRAUMA | 16 | 17 | 2 | 837 | 17 | 2018 | 17 | Q2 | 3.8 |
| **3** | CELLS | 14 | 18 | 2 | 1020 | 18 | 2019 | 18 | Q2 | 5.2 |
| **4** | NEURAL REGENERATION RESEARCH | 13 | 24 | 1.444 | 621 | 24 | 2017 | 24 | Q2 | 6.7 |
| **5** | FRONTIERS IN CELLULAR NEUROSCIENCE | 12 | 13 | 1.2 | 943 | 13 | 2016 | 13 | Q3 | 4 |
| **6** | MOLECULAR NEUROBIOLOGY | 11 | 17 | 1.375 | 491 | 17 | 2018 | 17 | Q2 | 4.3 |
| **7** | FRONTIERS IN NEUROSCIENCE | 9 | 12 | 1 | 669 | 12 | 2017 | 12 | Q3 | 3.2 |
| **8** | PHARMACEUTICS | 9 | 9 | 1.5 | 334 | 9 | 2020 | 9 | Q3 | 5.5 |
| **9** | BIOMOLECULES | 8 | 8 | 1.333 | 332 | 8 | 2020 | 8 | Q2 | 4.8 |
| **10** | EXPERIMENTAL NEUROLOGY | 8 | 8 | 1 | 270 | 8 | 2018 | 8 | Q2 | 4.2 |
| **11** | FRONTIERS IN IMMUNOLOGY | 8 | 9 | 1.143 | 225 | 9 | 2019 | 9 | Q2 | 5.9 |
| **12** | FRONTIERS IN MOLECULAR NEUROSCIENCE | 7 | 9 | 0.875 | 347 | 9 | 2018 | 9 | Q3 | 3.8 |
| **13** | FRONTIERS IN NEUROLOGY | 7 | 11 | 0.875 | 205 | 11 | 2018 | 11 | Q3 | 2.8 |
| **14** | SCIENTIFIC REPORTS | 7 | 8 | 1.4 | 129 | 8 | 2021 | 8 | Q3 | 3.9 |
| **15** | FRONTIERS IN AGING NEUROSCIENCE | 6 | 8 | 1.2 | 89 | 8 | 2021 | 8 | Q3 | 4.5 |

**Supplementary Table 5.** Top 10 Most Cited Publications Based on Bibliometrix Analysis

| **Rank** | **Author(s)** | **Title** | **Journal** | **Year** | **DOI** | **TC** | **TC/yr** | **Norm TC** |
| --- | --- | --- | --- | --- | --- | --- | --- | --- |
| 1 | Kalluri R | The biology, function, and biomedical applications of exosomes | Science | 2020 | 10.1126/science.aau6977 | 7821 | 1303.50 | 42.99 |
| 2 | Zhang Y, Chopp M, Meng Y, Katakowski M, Xin H, Mahmood A, Xiong Y | Effect of exosomes derived from multipluripotent mesenchymal stromal cells on functional recovery and neurovascular plasticity in rats after traumatic brain injury | J Neurosurg | 2015 | 10.3171/2014.11.JNS14770 | 578 | 52.55 | 2.99 |
| 3 | Zhang ZG, Buller B, Chopp M | Exosomes -- beyond stem cells for restorative therapy in stroke and neurological injury | Nat Rev Neurol | 2019 | 10.1038/s41582-018-0126-4 | 432 | 61.71 | 5.05 |
| 4 | Wang KK, Yang Z, Zhu T, Shi Y, Rubenstein R, Tyndall JA, Manley GT | An update on diagnostic and prognostic biomarkers for traumatic brain injury | Expert Rev Mol Diagn | 2018 | 10.1080/14737159.2018.1428089 | 422 | 52.75 | 4.20 |
| 5 | Hade MD, Suire CN, Suo Z | Mesenchymal Stem Cell-Derived Exosomes: Applications in Regenerative Medicine | Cells | 2021 | 10.3390/cells10081959 | 381 | 76.20 | 8.14 |
| 6 | Kim DK, Nishida H, An SY, et al. | Chromatographically isolated CD63+CD81+ extracellular vesicles from mesenchymal stromal cells rescue cognitive impairments after TBI | Proc Natl Acad Sci USA | 2016 | 10.1073/pnas.1522297113 | 378 | 37.80 | 2.35 |
| 7 | Tan F, Li X, Wang Z, Li J, Shahzad K, Zheng J | Clinical applications of stem cell-derived exosomes | Signal Transduct Target Ther | 2024 | 10.1038/s41392-023-01704-0 | 364 | 182.00 | 24.98 |
| 8 | Szepesi Z, Manouchehrian O, Bachiller S, Deierborg T | Bidirectional microglia-neuron communication in health and disease | Front Cell Neurosci | 2018 | 10.3389/fncel.2018.00323 | 356 | 44.50 | 3.54 |
| 9 | Zhang Y, Chopp M, Zhang ZG, Katakowski M, Xin H, Qu C, Ali M, Mahmood A, Xiong Y | Systemic administration of cell-free exosomes generated by human bone marrow derived mesenchymal stem cells cultured under 2D and 3D conditions improves functional recovery in rats after traumatic brain injury | Neurochem Int | 2017 | 10.1016/j.neuint.2016.08.003 | 314 | 34.89 | 2.80 |
| 10 | Gupta D, Zickler AM, El Andaloussi S | Dosing extracellular vesicles | Adv Drug Deliv Rev | 2021 | 10.1016/j.addr.2021.113961 | 295 | 59.00 | 6.30 |
